# Effect of correlating adjacent neurons for identifying communications: Feasibility experiment in a cultured neuronal network

**DOI:** 10.3934/Neuroscience.2018.1.18

**Published:** 2017-12-25

**Authors:** Yoshi Nishitani, Chie Hosokawa, Yuko Mizuno-Matsumoto, Tomomitsu Miyoshi, Shinichi Tamura

**Affiliations:** 1 Department of Radiology, Graduate School of Medicine, Osaka University, Suita 565-0871, Japan; 2 Biomedical Research Institute and Advanced Photonics and Biosensing Open Innovation Laboratory, AIST, Ikeda, Osaka 563-8577, Japan; 3 Graduate School of Applied Informatics, University of Hyogo, Kobe 650-0044, Japan; 4 Department of Integrative Physiology, Graduate School of Medicine, Osaka University, Suita 565-0871, Japan; 5 NBL Technovator Co., Ltd., Sennan 590-0522, Japan

**Keywords:** cultured neuronal network, spike wave propagation, adjacent neurons, identifying communications, microelectrode array

## Abstract

Neuronal networks have fluctuating characteristics, unlike the stable characteristics seen in computers. The underlying mechanisms that drive reliable communication among neuronal networks and their ability to perform intelligible tasks remain unknown. Recently, in an attempt to resolve this issue, we showed that stimulated neurons communicate *via* spikes that propagate temporally, in the form of spike trains. We named this phenomenon “*spike wave propagation*”. In these previous studies, using neural networks cultured from rat hippocampal neurons, we found that multiple neurons, *e.g.*, 3 neurons, correlate to identify various spike wave propagations in a cultured neuronal network. Specifically, the number of *classifiable neurons* in the neuronal network increased through correlation of spike trains between current and adjacent neurons. Although we previously obtained similar findings through stimulation, here we report these observations on a physiological level. Considering that individual spike wave propagation corresponds to individual communication, a correlation between some adjacent neurons to improve the quality of communication classification in a neuronal network, similar to a diversity antenna, which is used to improve the quality of communication in artificial data communication systems, is suggested.

## 1. Introduction

The brain is a well-known large neuronal network assembled through spike propagation (action potentials) through synapses [1]–[5]. How can neuronal networks comprising neurons with fluctuating characteristics reliably communicate (transmit information)? Many previous studies have attempted to answer this question, using spike-coding metrics [6], spatiotemporal coding models [7]–[13], and synchronous action models [14]–[18]. Since neuronal networks are considered spatiotemporal spike propagation fields and since the spatiotemporal form of spike activity is considered the fundamental generator of intelligence in the brain, these studies primarily aimed to investigate the principles of spike propagation in detail; however, these studies could not elucidate the basic means of communication between neurons. Therefore, the mechanisms underlying communication in the brain remain unknown.

In our previous studies attempting to resolve this issue, we reported that spikes propagating from stimulated neurons are received by afferent neurons as random-like sequences in simulated and natural asynchronous neuronal networks [19]–[24]. This phenomenon is similar to radio wave propagation in artificial data communication systems; hence, this phenomenon was referred to as “spike wave propagation.” In these studies, we showed that stimulated neurons were able to identify various spatiotemporal patterns of spike wave propagation in specific areas (receiving area) of the neuronal network. From the viewpoint of communication, individual spike waves propagating from specific neurons are regarded as individual communication, thereby suggesting that distinct communications occur in multiple brain neuronal networks. In addition, certain adjacent neurons correlate to classify communications, in simulated neuronal networks [23].

On in-depth investigation, numerous neurons seem involved in communication, *e.g.*, 3 neurons in the receiving area, and result in smooth and stable spike propagation, whereas fewer neurons make communication more difficult. This suggests a correlation between some adjacent neurons to improve the classification quality of communication in neuronal networks, similar to diversity antennae, used to improve the quality of communication in artificial data communication systems [25].

Although this observation was exclusive to simulation studies, remarkable similarities in the manner of correlation in some adjacent neurons for intelligence activity have been observed [26]. If these similarities hold true, they could provide evidence to determine the mechanism underlying communication in the brain. To accomplish this, we need to investigate whether the same phenomenon is observed physiologically as with simulation. Thus, this study aimed to investigate this phenomenon in a cultured neuronal network and determine if its physiological correlates are in line with those demonstrated in previous simulation studies.

## 2. Materials and method

### 2.1. Coding spike trains from a cultured neuronal network

**Figure 1.**
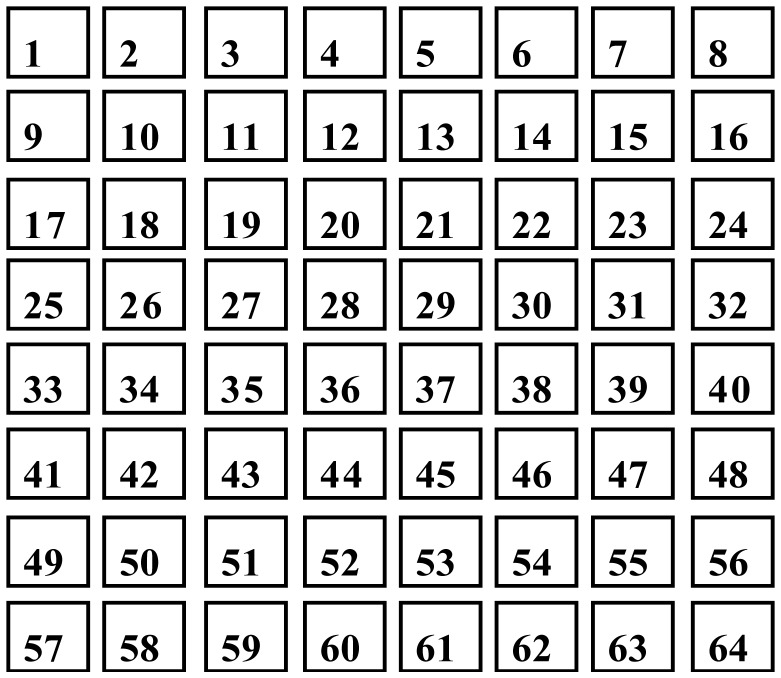
Deposition of electrodes in a microelectrode array. The size and spacing between electrodes were 50 × 50 µm^2^ and 150 µm (MED-P515A) or 450 µm (MED-P545A), respectively. Each electrode corresponds to a channel of spike recording. The number indicates the electrode (channel) number.

Hippocampal neurons were dissected from Wistar rats on embryonic day 18. The procedure conformed to the protocols approved by the Institutional Animal Care and Use Committee of the National Institute of Advanced Industrial Science and Technology. Cell culturing, stimulated spike recording, and coding spike trains were performed as described previously [24]. Figure 1 depicts the deposition of an electrode in microelectrode array (MEA) dishes with 64 (8 × 8) planar microelectrodes (channels). In this study, we cultured 5 samples of neuronal networks named cultures 01, 02, 03 planted on MED-P515A (spacing between electrodes, 150 µm) and cultures 04 and 05 planted on MED-P545A (spacing between electrodes, 450 µm). Two channels in each culture were selected as stimulation channels and 5–20 recordings were obtained from them. In this study, the stimulated channels are referred to as StimA and StimB. This experiment aimed to classify the StimA and B in accordance with time sequence data based on spike trains in specific neurons (current channels) and their adjacent neurons. The difference in stimulation channel was regarded as the difference of spike wave propagation [24].

### 2.2. Time sequence data with spike timing lag from adjacent channel

**Figure 2.**
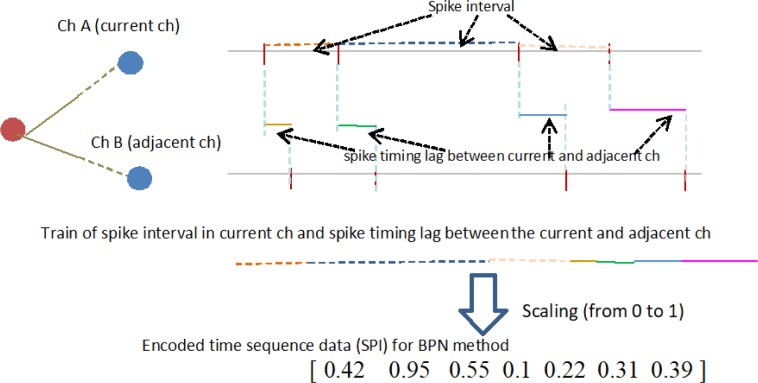
Procedure of generating time sequence data. The dashed color lines represent spike intervals in the current channel. Solid color lines are spike timing lags between the current and their adjacent neurons. The sequence data are spike train intervals in the current channel followed by the time lag in spike train intervals between current and adjacent channels. These time sequence data are encoded for the BPN method.

**Figure 3.**
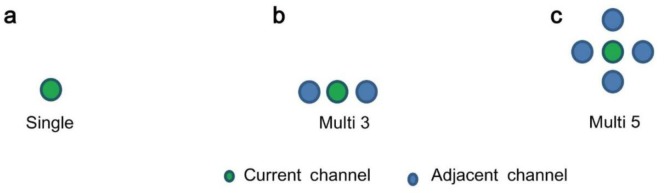
Location of adjacent channel. a: Without adjacent channels (only current channel) named *Single*; b: With right and left adjacent neurons named *Multi 3*; c: With right, left, upper, and lower adjacent neurons named *Multi 5*.

To estimate improvement in the classification quality of communication through correlation of adjacent neurons, as shown in Figure 2, we constructed time sequence data which linked the spike train interval of current channels (ch) with the time lag in spike train intervals between current and adjacent channels. Although Figure 2 shows only one adjacent channel for simplicity, several adjacent channels were actually referred. We compared the time sequence based on three types of adjacent channel locations, as shown in Figure 3. Moreover, time differences from the current channel are signs of delayed (+1) or preceding (−1) spike trains; however, this figure shows only delayed spike trains. Further, the encoded time sequence was generated from these time sequence data for the back propagation of neuronal networks (BPN) method described below in detail. These procedures were performed for all 64 channels in each culture.

### 2.3. Classification by BPN method

On fluctuating conditions in neuronal networks, *e.g.*, synaptic weight, refractory period, and others, spatiotemporal patterns of spike wave propagations were not the same even if they were stimulated at the same channel in the same culture. Therefore, identifying the stimulated channels by only observing wave propagation is not easy. In our previous study, we used our original simple learning algorithm based on the arithmetic mean method for classification [24]. However, the resolution was not adequate to classify spike wave propagations completely. Thus, we believe that the BPN method has moderately strong pattern recognition ability [27] and could hence be applied to show the feasibility of classifying spatiotemporal patterns of spike wave propagation in neuronal networks, in this study.

**Figure 4.**
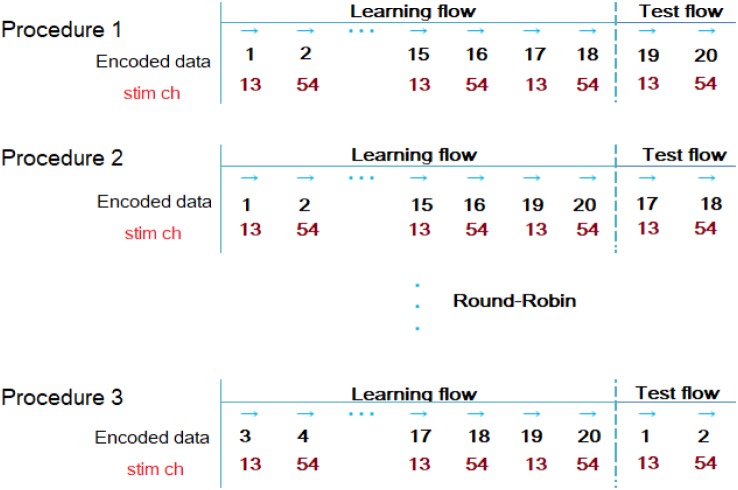
Round-Robin learning and test procedure for back propagation of neuronal networks (in culture 01). The example depicted here is of the encoded data number and stimulation channel (stim ch) number in the case of culture 01 (stim ch 13 *vs* 54). Twenty raster plot data (10 raster plot data stimulated from channel ch13 and 10 raster plot data stimulated from channel ch54) are encoded by the procedure shown in Figure 2; among them, two encoded data were selected for *Test Flow* and another 18 data were used for *Learning Flow*. Finally, all data were tested using the Round-Robin method.

Round–Robin learning and the test procedure for the BPN method are depicted in Figure 4. This figure depicts the example of the encoded data number and stimulation channel (stim ch) number in the case of culture 01 (stim ch 13 *vs* 54).

### 2.4. Estimation of the classification quality

To estimate the effect of correlation between current and adjacent channels and the classification quality, we calculated the rate of test data wherein the stimulation channel was detected correctly at the current channel in the BPN procedure; this was called “success rate ch” and the rate of test data wherein the stimulation channel was detected incorrectly (for example, in culture 01, the detection result was st54 when the real stimulating channel was channel 13) at current channel called fail rate ch. The equations for determining the success rate ch and fail rate ch are as follows:

$${\text{success rate}}_{ch}=\frac{dtcablenum_{ch}}{testnum}\times 100[\%]$$ (1)

$${\text{fail rate}}_{ch}=\frac{eronum_{ch}}{testnum}\times 100\left[\%\right]$$ (2)

In these equations, *dtcablenum_ch_* is the number of test data when the stimulation channel was detected correctly at the current channel, *test num* is the number of test data (5 to 10), *eronum_ch_* is the number of test data when the stimulation channel was detected incorrectly. In this study, we considered the *success rate_ch_* > 60% as the *classifiable channel*. In other words, two different stimulations could correctly identify the *classifiable channel*. Furthermore, we regarded *fail rate_ch_* > 60% as the *miss-classifiable channel*.

## 3. Results and discussion

**Figure 5.**
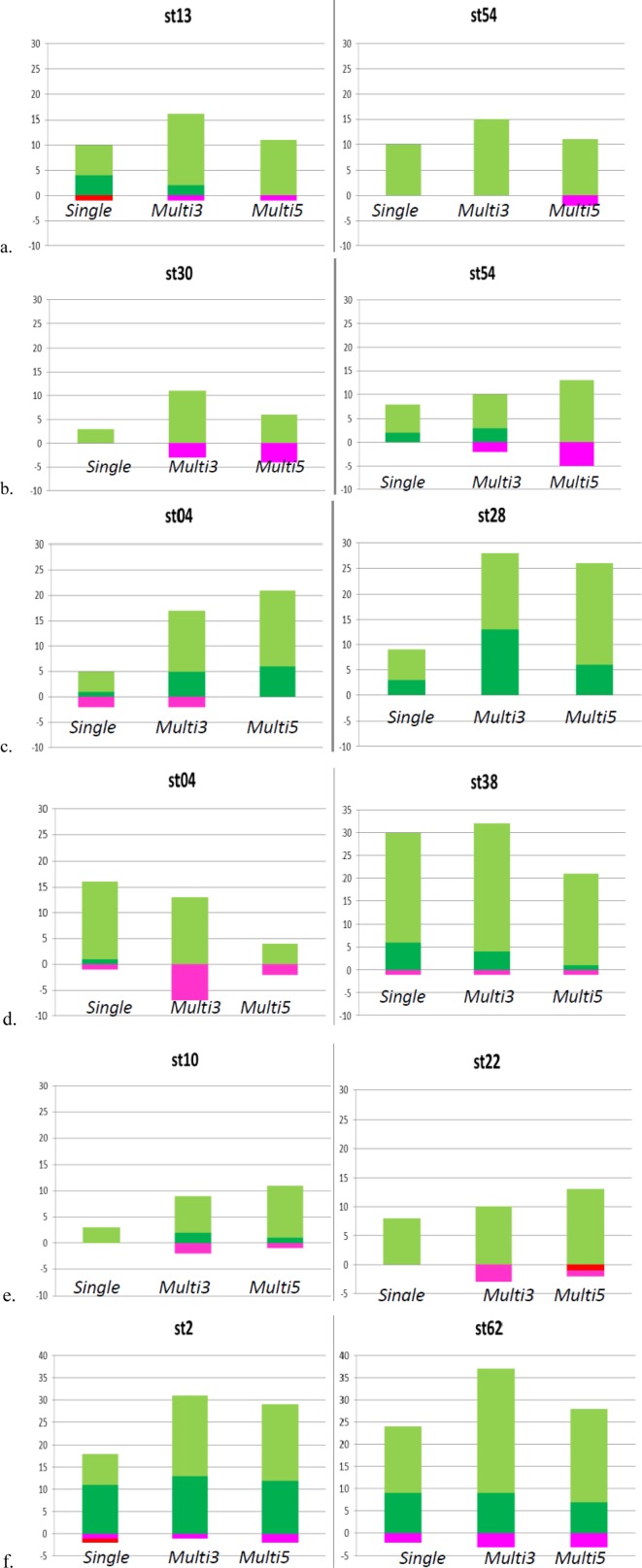
The number of classifiable channels in 64 channels in each culture.a: Culture 01 stimulation channel 13 *vs* 54; b: Culture 01 stimulation channel 30 *vs* 54; c: Culture 02 stimulation channel 04 *vs* 28; d: Culture 03 stimulation channel 04 *vs* 38; e: Culture 04 stimulation channel 10 *vs* 22; f: Culture 05 stimulation channel 2 *vs* 62. Vertical axis: Number of *classifiable channels* (positive) and *miss-classifiable channels* (negative); St: Stimulating channel. 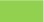
 Classifiable channels the stimulated neuron was detected correctly in 60%–80% of probability. 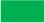
 Classifiable channels the stimulated neuron was detected correctly in 80% of probability or more. 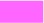
 Miss-Classifiable channels the stimulated neuron was detected incorrectly in 60%–80% of probability. 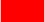
 Miss-Classifiable channels the stimulated neuron was detected incorrectly in 80% of probability or more.

Figure 5 shows the number of *classifiable channels* and *miss-classifiable channels* in 64 channels in each culture in *Single*, *Multi3*, and *Multi5* (Figure 3). Though, in some cases, the number of *classifiable* channels is smaller in *Multi* than in *Single*, contrary to our assumption similar to that in Culture 03, we could confirm that the mean number of classifiable channels in all experiments in *Multi3* and *Multi5* was significantly larger than that in *Single* (*p* < 0.05). However, the difference between *Multi* *5* and *Multi 3* was not significant; furthermore, *Multi 5* tended to be worse than *Multi 3*. Meanwhile, the number of *miss-classifiable channels* tended to be larger in *Multi* than in *Single*; however, in reality, they were fewer than the number of *classifiable channels*. These results show that the time lag in spike trains between adjacent neurons may effectively improve the quality of communication.

**Figure 6.**
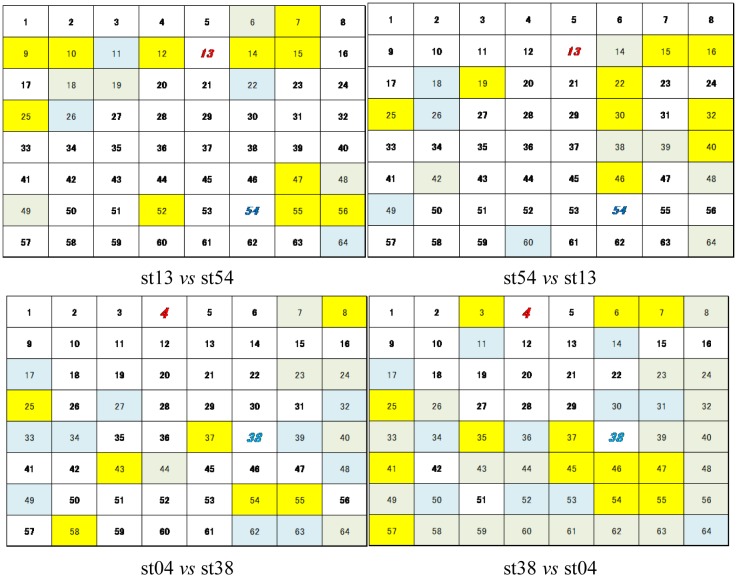
Distribution of classifiable channels. Each lattice and lattice number indicate a channel and channel number, respectively. Lattice numbers written in ***colored (red/blue) bold italic type*** indicate stimulating neurons. 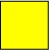
 Classified in *Multi3* only 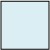 Classified in *Single* only 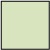 Classified in both *Single* and *Multi3*

In these experiments, we could confirm the increase in the rate of classification through the schematic correlation of adjacent neurons. However, it remained unclear which neurons are classifiable by correlating adjacent neurons. Hence, we compared the distribution of classifiable channels in neuronal networks in *Single* and mul3 in Culture 1 and 3, as examples (Figure 6). We found that in culture 1, some unclassifiable channels in *Single* were classifiable in *Multi3* (indicated by yellow lattices in Figure 6). Moreover, these channels tended to concentrate in specific areas of the neuronal network. These tendencies were also observed in culture 3 despite the number of classifiable channels in *Multi3* being no more than that in *Single*, as shown in Figure 5(d). However, channels that classified only in *Single* (indicated by sky blue lattices in Figure 6) were also observed at similar numbers to that of the classifiable channels only in *Multi3* in culture 03. In contrast, in culture 01, the number of channels which classified only in *Single* was lower than those that became classifiable in *Multi3*. The difference between these results in Figure 5(a) and (d) corresponded to this. However, spacing between electrodes did not affect the experimental results (150 µm: culture 01, 02, and 03; 450 µm: culture 04 and 05).

**Figure 7.**
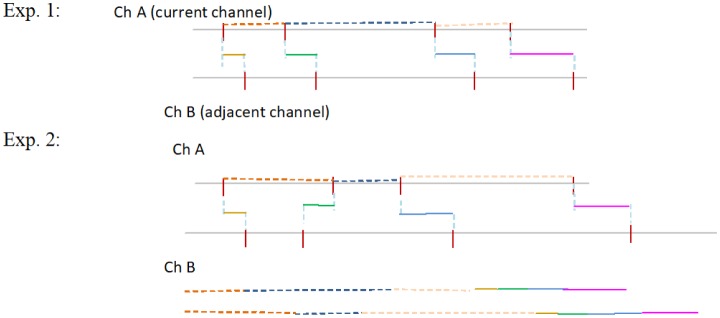
A case wherein correlating the adjacent neuron was effective. The meaning of the culler horizonal line and dashed culler horizonal line is the same as that in Figure 2.

**Figure 8.**
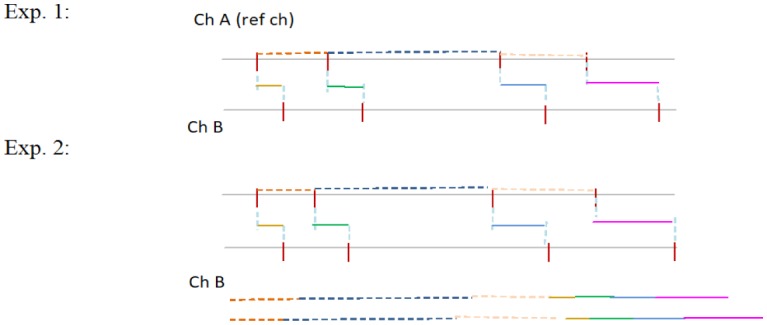
A case wherein correlating the adjacent neuron was not effective. The meaning of the culler horizonal line and dashed culler horizonal line is same as that in Figure 2.

We considered the following reasons for these results. If various spike interval trains of trials are large even in the same stimulation channel, it is difficult to detect the stimulation channel of only the spike interval train in the current channel. However, if the time lag in the spike trains between the current channel and the adjacent channel does not display variety, stimulated neurons could only be detected from the train of the time lag. The image so obtained is shown in Figure 7. In this case, the stimulation channel is detectable from the difference in spike timing between the current channel and the adjacent channel. When spike interval trains in all experiments in the current channel did not display variety, only the spike train intervals in the current channel were adequate to detect the stimulation channel. The image so obtained is shown in Figure 8. In this case, it is possible that the rate of classification is worse based on the variety of spike trains in the adjacent channel.

**Figure 9.**
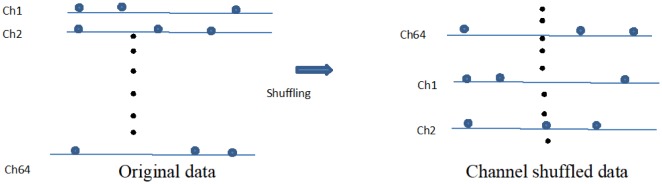
Channel shuffling. The row of channels was shuffled randomly, retaining the spike trains in each channel.

To substantiate these assumptions, we decorrelated the current channel and adjacent channel by *channel shuffling* for all cultured samples; the procedure followed is shown in Figure 9. Thereafter, we performed the same experiments for the shuffled data, as for the original data.

**Figure 10.**
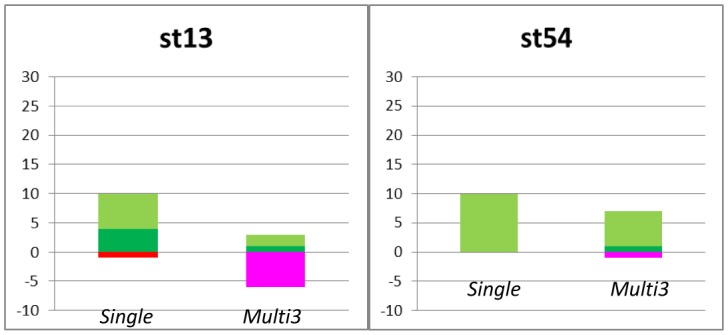
The number of detectable channels in 64 channels after ch shuffling in culture 01. Vertical axis: Number of *classifiable channels* (positive) and *miss-classifiable channels* (negative). st: Stimulating channel.

**Figure 11.**
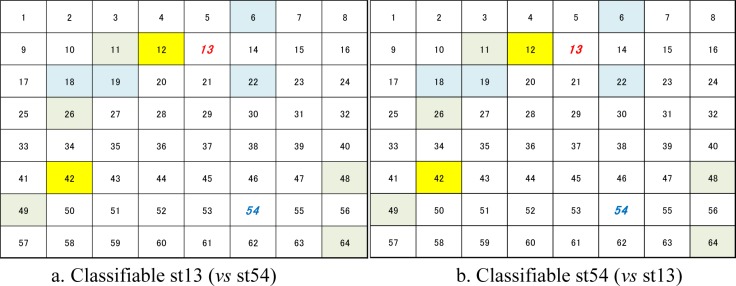
Distribution of classifiable channels after ch shuffling in culture 01(st13 *vs* 54). Each lattice and lattice number indicates the channel and channel number, respectively. Lattice numbers written in colored (red/blue) bold italic type indicate stimulating neurons. 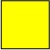
 Classified in *Multi3* only 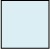 Classified in *Single* only 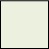 Classified in both *Single* and *Multi3*

Figure 10 shows the number of *classifiable channels* and *miss-classifiable channels*, and Figure 11 depicts the example of the distribution of *classifiable channels* in neuronal networks in *Single* and *Multi3* for *channel shuffled data* of culture 01. Although data from other cultures are not shown owing to lack of space, results from these cultures were similar to those from culture 01, as described below. In this experiment, the detection of classifiable channels was performed only with *Multi3*, since it is adequate to determine the effect of neurons if this effect is observable in *Multi3* or *Multi5*; however, it is considered that the results after channel shuffling remain the same as before channel shuffling in *Single*.

*Multi3* mean values were remarkably lower than those of *Single*; however, there was no significant difference between their mean values in all cultures.

Based on the results from all cultures in this experiment, we could confirm that the number of *classifiable channels* in the shuffled data was significantly (*p* < 0.05) lower than that in the original data in *Multi3* (Cf. Figure 10 *vs* Figure 5(a), and Figure 11 *vs* Figure 6(a)). However, the difference between *Multi3* and *Single* was not significant in the shuffled data, although the values in *Multi3* were lower than those in *Single* in several cultures including culture 01 (Figure 10), against those in the original data.

In summary, we could confirm the effect of spike timing lag between the current and their adjacent neurons. The higher number of *classifiable channel*s in *Multi3,5* than in *Single* was not a chance event. Considering that individual stimulation channels correspond to individual communication, in this study, although the classification quality of communications was not always improved, various communications could be characterized by the correlating spike trains of adjacent neurons even when they could not be characterized by only the current neuron.

## 4. Conclusion

In our recent study, we showed that stimulated neurons could identify various spatiotemporal patterns of spike wave propagation in particular areas of neuronal networks. From the viewpoint of communication, this essentially suggests that distinct communications occur *via* multiple communicating links in the brain. Here, we showed that the quality of communication classification tends to improve *via* correlation of spike trains in current and their adjacent neurons. This shows that neighboring neurons work in harmony to identify communication. Assuming a communication path is a type of memory, it seems that the present results are concurrent with previous ones, indicating that some adjacent neurons work in harmony; however, this requires more detailed investigation.
